# Global burden and trends of norovirus-associated diseases from 1990 to 2021 an observational trend study

**DOI:** 10.3389/fpubh.2024.1483149

**Published:** 2025-01-07

**Authors:** MengLan Zhu, ZiLing Huang, TongTong Liu, ChenNan Wu, ZhiHan Shang, LuLu Zhang

**Affiliations:** ^1^Sanitation Teaching and Research Section, Department of Health Service, Naval Medical University, Shanghai, China; ^2^Otolaryngology Department of Unit 32265 of the People’s Liberation Army, Guangzhou, China; ^3^Department of the 4th Accreditation Outpatient, General Hospital of the Southern Theater of the Chinese People’s Liberation Army, Guangzhou, China; ^4^Department of Neurology, The 305th Hospital of the People’s Liberation Army, Beijing, China

**Keywords:** norovirus-associated diseases, global burden of diseases, mortality rate, disability-adjusted life years, global trend analysis

## Abstract

**Background:**

Norovirus remains a significant viral cause of waterborne and foodborne gastroenteritis outbreaks and epidemics worldwide. The burden of norovirus extends across different income settings.

**Methods:**

Leveraging secondary data from the 2021 Global Burden of Diseases Study, our analysis spanned the period from 1990 to 2021 to assess the burden of norovirus-associated diseases (NADs). We utilized descriptive statistics to examine global mortality rates and disability-adjusted life years (DALYs). For trend analysis, we employed annual percentage change (EAPC) through linear regression and applied Joinpoint analysis to identify significant changes over time. A comprehensive age-period-cohort model evaluated the key mortality risk factors. Furthermore, a Bayesian age-period-cohort analysis was conducted to forecast trends up to 2035, providing valuable insights for policy formulation and resource allocation.

**Results:**

In 2021, the global age-standardized mortality rate (ASMR) for NADs was 1.62 per 100,000 population (95% UI: 0.35 to 2.91), while the age-standardized DALY rates (ASDR) was 79.02 years per 100,000 population (95% UI: 26.61 to 132.26). A downward trend was observed in most regions and countries, with EAPC of −4.29% (95% UI: −4.53 to −4.05) for ASMR and −4.40% (95% UI: −4.62 to −4.19) for ASDR from 1990 to 2021. Notably, children under 5 years old had considerably higher ASDR: 475.52 years (95% UI: 160.73 to 893.72) per 100,000 for males and 335.44 years (95% UI: 112.29 to 623.48) per 100,000 for females. Mortality risk from NADs escalated with age, with a peak of 69.27 (95% CI: 64.04 to 74.92) per 100,000 for the under-five age group, and 11.38 (95% CI: 10.59 to 12.22) per 100,000 for individuals over 95 years. Bayesian Age-Period Cohort projections indicate a continued decline in ASMR and ASDR through 2035.

**Discussion:**

Between 1990 and 2021, ASMR and ASDR for NADs significantly declined due to public health interventions, vaccination, and improved sanitation. However, norovirus remains highly contagious, especially among children and the older adult. Projections suggest a continued decrease in the burden of such diseases by 2035. To further reduce this burden, preventive measures like vaccination and infection control strategies are essential for high-risk populations, alongside ongoing research into norovirus epidemiology and transmission dynamics.

## 1 Introduction

Norovirus is a leading cause of diarrheal diseases, accounting for 18% of global cases and imposing an economic burden of approximately $60 billion worldwide (1, 2). It poses a significant public health challenge and economic burden worldwide (3), impacting individuals of all ages and contributing substantially to the global disease burden (4). Recent research has shed light on the distribution of norovirus-associated diseases (NADs) within populations, host susceptibility factors, and case ascertainment methodologies, deepening our understanding of norovirus epidemiology (5).

Young children bear the highest incidence of NADs, with severe outcomes particularly affecting this age group and the older adult. Children under five have an infection rate of 18% (6, 7), highlighting the need for targeted control interventions, including vaccination strategies (8). The older adult, especially those over 80, face an increased risk of norovirus infection and hospitalization, leading to longer hospital stays and higher costs (9).

Geographic variation in norovirus prevalence is substantial, with higher population densities correlating with increased prevalence (10). The burden of NADs in different countries is influenced by factors such as healthcare access, urbanization, and education levels, which shape the epidemiological landscape and impact disease prevalence and healthcare systems (11). Higher urbanization can exacerbate disease transmission and healthcare demands, particularly in urban populations (12).

Although global mortality rates and disability-adjusted life years (DALYs) related to NADs have significantly decreased, their public health impact remains considerable (11). Significant gaps exist in current research, including a lack of detailed epidemiological data analysis for specific regions globally, missing studies on temporal trend variations, insufficient systematic summaries of high-risk populations, and inadequate research on future disease burden trend predictions. These gaps limit our comprehensive understanding of the impact of norovirus and affect the development of effective prevention strategies. This study aims to assess the disparities in NADs’ mortality and DALYs across regions and demographic groups over the past 31 years and project these trends through 2035, to inform targeted public health interventions.

## 2 Methods

### 2.1 Material

Our study utilized data exclusively from the Institute for Health Metrics and Evaluation (IHME) website,^1^ specifically employing the Global Burden of Disease (GBD) result tool to extract “Norovirus”-related mortality and DALYs data under the “etiology” category.

The IHME’s Bayesian regression tool, DisMod-MR 2.1, was applied for the analysis, modeling, and estimation of these indicators, standardizing them for global populations and reporting them as age-standardized rates of mortality (ASMR) and DALYs (ASDR) per 100,000 people. All estimates are presented with 95% uncertainty intervals (UIs), accounting for all uncertainties due to measurement errors, biases, and modeling. The 95% UIs are derived from the 2.5th and 97.5th percentiles of 1,000 samples (13).

The covariates applied to the mortality and DALY indicators for norovirus in this database can be reviewed in the appendices of published GBD 2021 papers, and the underlying raw data are accessible through a searchable online tool.^2^

Case ascertainment involved recruiting sentinel hospitals, collecting stool samples, screening for norovirus using qRT-PCR, confirming positive samples with conventional RT-PCR (14, 15). The GBD study employed a counterfactual approach to estimate the etiological fraction of diarrheal diseases attributable to norovirus. This involved calculating a population attributable fraction (PAF) based on the proportion of severe diarrhea cases positive for norovirus. The PAF indicates the potential reduction in diarrheal mortality in the absence of norovirus. The formula used to estimate PAF is:PAF=Proportion*(1-1/OR),where Proportion is the fraction of diarrhea cases that test positive for norovirus, and OR is the odds ratio for diarrhea associated with the presence of norovirus (16).

Some results are stratified by the Socio-demographic Index (SDI), a composite measure reflecting income distribution, average years of education, and fertility rates for women under 25 (15). The global population and SDI data utilized in this study were sourced from the GBD website.^3^

### 2.2 Descriptive analysis

Descriptive analyses were conducted at the global, regional, and national levels. From 1990 to 2021, the global number of mortality and DALYs, ASMR and ASDR were visually presented for both sexes, males, and females. Additionally, comparisons of the number and age-standardized rates (ASR) of mortality and DALYs between 1990 and 2021 were made across the global, regions (21 GBD geographical regions), countries (204 countries and territories), and the five SDI quintiles.

### 2.3 Trend analysis

We aim to explore the development trends of NADs on a global, regional, and national scale. First, we used the Estimated Annual Percentage Change (EAPC) to quantify the overall trend in the burden of NADs (17). The linear regression equation was used to calculate EAPC of mortality and diseases burden and ASDR between the whole world from 1990 to 2021, and to analyze its change trend (18). The linear regression model:


y=α+βx

*Where y = ln (ASR)* and x represents the years. The EAPC is then calculated using the formula:


$$\mathrm{EAPC}\;=\;\left(\exp\left(\beta \right)\;-\;1\right)*\;100\%$$

We use Joinpoint regression analysis^4^ to detect local trends in the burden of NADs. Joinpoint regression analysis can divide the overall trend into multiple segments based on the Joinpoints, and by calculating the Annual Percentage Change (APC) for each segment and its 95% CI, it further evaluates the magnitude of each trend. If the APC estimate and the lower bound of its 95% CI are both greater than 0, the trend is considered to be increasing during that period. Conversely, if the APC estimate and the upper bound of its 95% CI are both less than 0, the trend is considered to be decreasing during that period. Otherwise, the trend is considered to be stable (17).

We analyzed the trends in the burden of NADs by evaluating age, period, and cohort effects. Given the complex interactions among these dimensions, an age-period-cohort (APC) model was utilized to estimate their distinct impacts on mortality risk. In this model, the age effect represents changes over an individual’s lifetime, the period effect encapsulates environmental influences affecting the entire population, and the cohort effect reflects variations experienced by cohorts born during the same period (19, 20). We used the Intrinsic Estimator (IE) method with Principal Component Regression Analysis and a Poisson distribution to model the equation:


$$\ln\left(Y_{i,j,k}\right)=\mu +\alpha_{i}+\beta_{j}+\gamma_{k}+\varepsilon_{i,j,k}$$

where *Y _i,j,k_* denotes the mortality rate in the *i*-th age group, *j*-th period, and *k*-th cohort; *μ* is the intercept; *α _i_*, *β _j_*,and *γ _k_* represent the age, period, and cohort effects, respectively; and *ε _i,j,k_* indicates the residual (21).

Data were organized into consecutive 5-year age groups (e.g., under 5, 5–9, …, 95+ years), 5-year periods from 1990 to 2021, and the corresponding 5-year birth cohorts (e.g., 1897–1901, …, 2017–2021) to estimate the net effects of age, period, and cohort on NADs mortality rates (22).

The APC model estimates the overall time trends and incidence trends within each age group. The former is represented as the annual percentage change in incidence, indicating the net drift formed by calendar time and successive birth cohorts (in percentage per year). The latter is represented as the annual percentage change in incidence by age, known as local drift (in percentage per year). The significance of the annual percentage change trends is assessed using the Wald *χ*2 test. Furthermore, in the APC model, the age effect is described by specific age rates consistent with birth cohorts, while the period/cohort effect is described as the relative risk of incidence associated with that period/cohort. This is calculated by comparing the age-specific rates for each period/cohort with the rates of a reference period/cohort. The choice of the reference period/cohort is arbitrary and does not affect the interpretation of the results (23).

### 2.4 Predictive analysis

We utilized the Bayesian Age-Period-Cohort (BAPC) model to forecast the global mortality changes of NADs from 2022 to 2035. The BAPC model, which takes a log-linear Poisson form, includes the effects of age, time, and cohort:


$$\log\left(yij\right)=\alpha +\mu i+\beta j+\gamma k\text{,}$$

where *α* is the intercept, *μi* represents the age effect, *βj* represents the period effect, and *γk* represents the cohort effect (24).

### 2.5 Statistics

In this study, our primary statistical analyses and chart generations were conducted using R Studio software (version 4.4.1). The R packages involved include ggmap (version 3.0.0) and GDAL (version 0.0.36), which were used for map plotting; BAPC (version 0.0.36), INLA (version 24.05.011), and ggplot2 (version 3.6.3) were utilized for more complex statistical analyses and chart generation. Additionally, the analysis of the Joinpoint model was performed using Joinpoint software (version 5.1.0), and the AAPC analysis was conducted using the online tool provided by the NIH official website at https://analysistools.cancer.gov/apc/. In all statistical analyses, *p* < 0.05 (two-tailed) was considered statistically significant.

## 3 Results

### 3.1 Temporal trend of NADs of global, regions, and countries from 1990 to 2021

**Figure 1:**
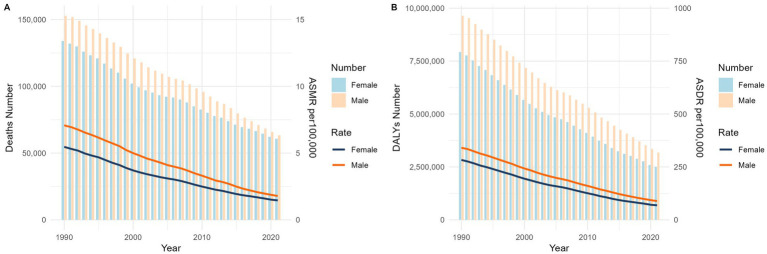
Global mortality **(A)** and disease burden **(B)** of Norovirus-Associated Diseases from 1990 to 2021. NADs, Norovirus-Associated Diseases; ASMR, age-standardized mortality rate; ASDR, age-standardized disability-adjusted life-years rate.

**Figure 2:**
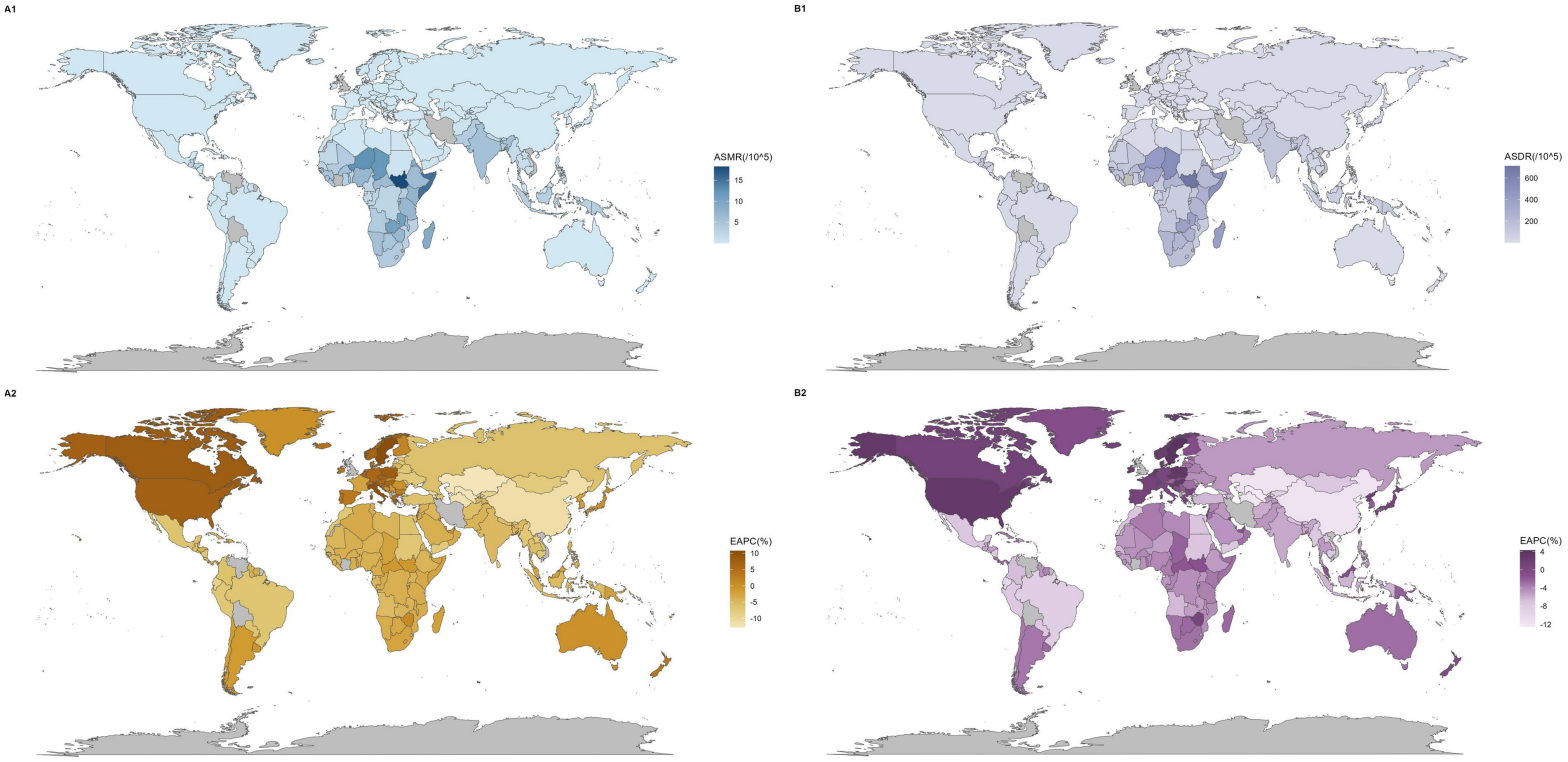
Global map of mortality and disease burden for NADs. The global diseases death **(A)** and burden **(B)** of NADs for both sexes in 198 countries and territories: (1) The ASMR and ASDR in 2021. (2) The trend in ASMR and ASDR (EAPC) from 1990 to 2021. NADs, Norovirus-Associated Diseases; ASMR, age-standardized mortality rate; ASDR, age-standardized disability-adjusted life-years rate; EAPC, estimated annual percentage change.

Figures 1, 2 demonstrate a significant decline in the global ASMR and ASDR for both sexes from 1990 to 2021. The ASMR decreased from 6.14 per 100,000 (95% UI: 1.49 to 10.63) to 1.62 per 100,000 (95% UI: 0.35 to 2.91), representing a statistically significant change (*p* < 0.001) with an EAPC of −4.29% (95% CI: −4.53 to −4.05).Countries with low SDI exhibited higher ASMR compared to those with high SDI. However, low-SDI countries experienced a more rapid decline, with an EAPC of −3.69% (95% CI: −3.93 to −3.45), while high-SDI countries showed an increase in ASMR with an EAPC of 2.95% (95% CI: 2.44 to 3.47).

From 1990 to 2021, the global ASDR significantly decreased, dropping from 310.88 (95% UI: 96.6 to 521.11) to 79.02 (95% UI: 26.61 to 132.26), with an EAPC of −4.40% (95% CI: −4.62 to −4.19). In contrast, high-SDI countries’ ASDR continued to increase, with an EAPC of 0.08% (95% CI: −0.17 to 0.33).

Figure 2 show variations in mortality and disease burden across countries. Most regions and countries saw declines in ASMR, but regions like Western Europe and high-income North America, and countries such as Sweden, Italy, and Canada, had an upward trend. Similarly, while DALY rates decreased in most regions and countries, regions including Western Europe and high-income North America, and countries like Sweden, Poland, and the Czech Republic, saw increases (Supplementary Tables S1, S2).

### 3.2 Descriptive analysis of the burden of NADs across all age groups for males and females globally in 2021

**Figure 3:**
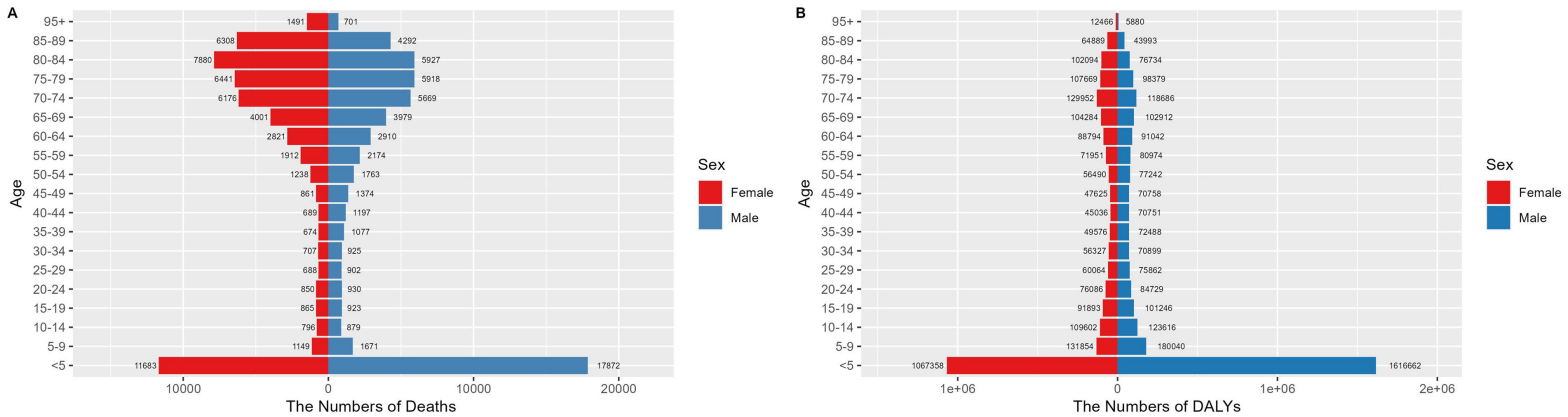
Number of deaths **(A)** and disease burden **(B)** from NADs across different age groups globally in 2021. NADs, Norovirus-Associated Diseases.

The under 5 age group has a significantly high concentration of deaths and DALYs. In this age bracket, there were 17,872 male deaths (95% UI: 5,799 to 33,813) and 11,683 female deaths (95% UI: 3,693 to 21,942). Males accumulated 1,616,662 DALYs (95% UI: 546,455 to 3,038,461), while females accumulated 1,067,358 DALYs (95% UI: 357,285 to 1,983,851). A paired t-test revealed no significant gender differences in mortality rates or disease burden (*p* > 0.05). For further details, refer to Figure 3 and Supplementary Tables S3.

**Figure 4:**
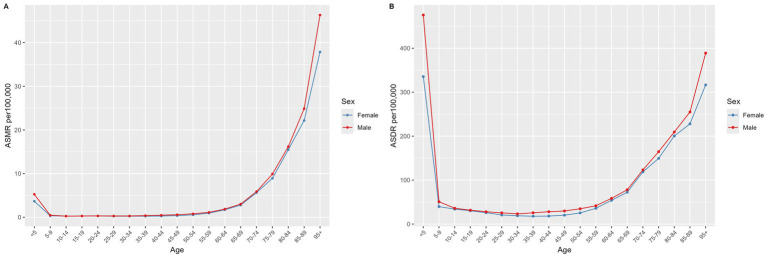
ASMR **(A)** and ASDR **(B)** from NADs across different age groups globally in 2021. NADs, Norovirus-Associated Diseases; ASMR, age-standardized mortality rate; ASDR, age-standardized disability-adjusted life-years rate.

Although the ASMR for children under five was comparatively low, with rates of 5.26 per 100,000 (95% UI: 1.71 to 9.95) for males and 3.67 per 100,000 (95% UI: 1.16 to 6.9) for females, the ASDR in this age group was considerably higher than in other age groups. Specifically, the ASDRs were 475.52 per 100,000 (95% UI: 160.73 to 893.72) for males and 335.44 per 100,000 (95% UI: 112.29 to 623.48) for females, as detailed in Figure 4.

### 3.3 Analyze the local trends of the burden of NADs using Joinpoint regression

**Figure 5:**
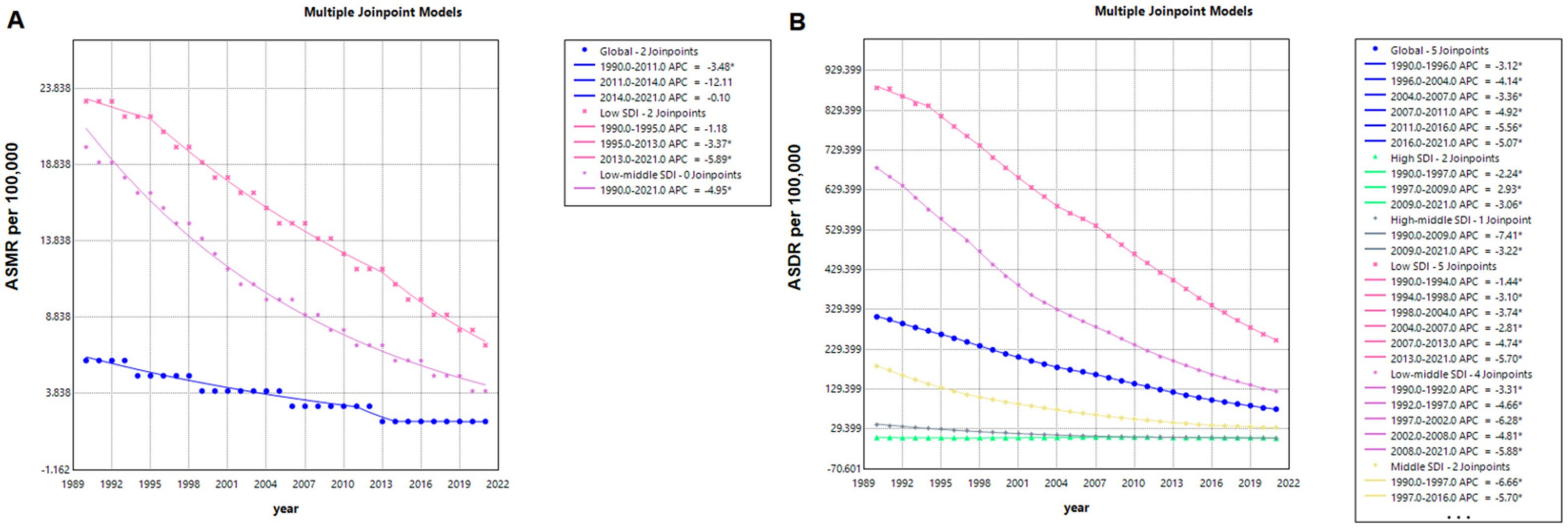
Joinpoint model of mortality **(A)** and disease burden **(B)** from NADs globally and in countries with different SDI levels from 1990 to 2021. NADs, Norovirus-Associated Diseases; ASMR, age-standardized mortality rate; DALYs, disability-adjusted life-years; ASR, age-standardized rate.

The Joinpoint regression analysis results, as displayed in Figure 5, reveal a continuous downward trend in the global ASMR from 1990 to 2021, with significant Joinpoints identified in 2011 and 2014. Notably, the period between 2011 and 2014 marked a particularly steep decline, with an APC of −12.11%. For countries with middle, high-middle, and high SDI, the ASMR values were too low to perform a Joinpoint regression analysis.

In the context of global ASDR for NADs, the analysis identified five Joinpoints at 1996, 2004, 2007, 2011, and 2016, with the steepest decline occurring from 2011 to 2016 (APC = −5.56%). When examining SDI categories, low SDI countries showed the most rapid decline in ASMR from 2013 to 2021 (APC = −5.89%), whereas high-middle SDI countries experienced the most significant ASDR reduction from 1990 to 2009 (APC = −7.41%).

### 3.4 Age-period-cohort analysis of mortality rates for NADs

**Figure 6:**
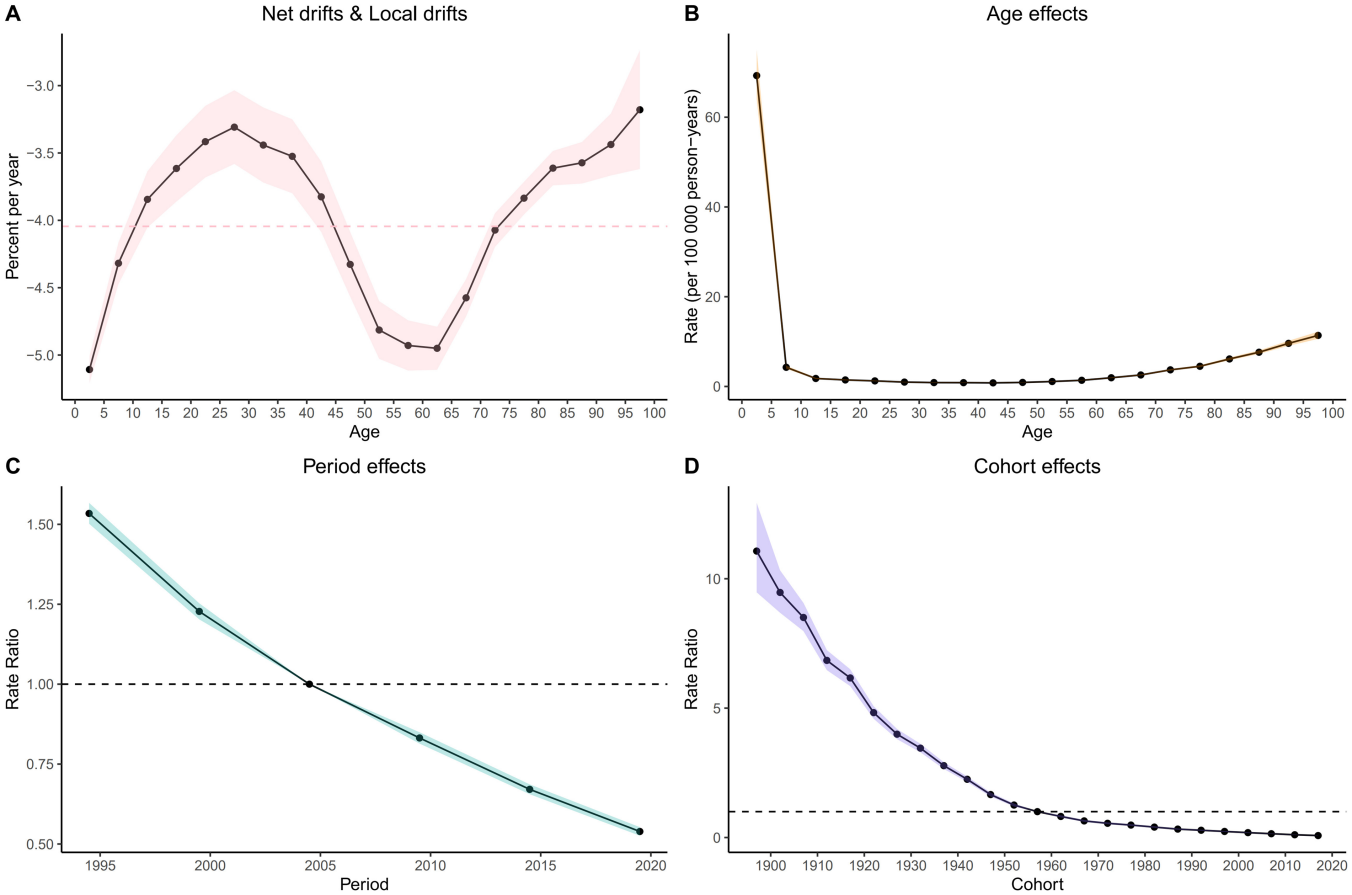
Local drift with net drift values **(A)** and The effects of age **(B)**, period **(B)**, and birth cohort **(D)** on the relative risk of NADs’ mortality.

Net drift refers to the overall annual percentage change in mortality rates across all age groups over the study period, while local drift indicates the annual percentage change in mortality rates specific to each age group relative to the net drift. The net drift of the ASMR for the global population is observed at - 3.83%. Among the age groups analyzed, local drift values were below zero, indicating a decrease in mortality rates, among which the most significant decrease is found in the 0–4 years age group (−5.11% per year), as demonstrated in Figure 6 and Supplementary Table S4.

After accounting for period and birth cohort effects, the age effect emerges as a significant determinant influencing the risk of mortality from NADs (Supplementary Figure S1; Supplementary Table S6). The mortality rate among global NADs patients initially declines steeply and then gradually increases over their lifespan. Notably, the mortality rate peaks at 69.27 (95% CI: 64.04 to 74.92) per 100,000 in the 0 to 4 years age group, decreases to a low of 0.8 (95% CI: 0.76 to 0.85) per 100,000 in the 40 to 44 years age group, and then rises to a maximum of 11.38 (95% CI: 10.59 to 12.22) per 100,000 for those aged 95 years and older.

Moreover, after a rigorous adjustment for age and birth cohort effects, a remarkable influence of the period effect on NADs mortality rates becomes evident. When compared with the period from 2002 to 2006, the period effect on the ASMR shows a consistent downward trajectory, with a relative risk (RR) of 1.53 (95% CI: 1.5 to 1.57) noted for the period from 1992 to 1996, which gradually decreases to an RR of 0.54 (95% CI: 0.53 to 0.55) for the years 2017 to 2021.

Besides, using the 1957 to 1961 cohort as a reference, the RR decreases from 2.27 (95% CI: 2.17 to 2.38) for individuals born in the 1895 to 1899 cohort to 0.32 (95% CI: 0.31 to 0.32) for the cohort born between 2017 and 2021.

### 3.5 Forecast analysis of the burden of NADs by 2035

**Figure 7:**
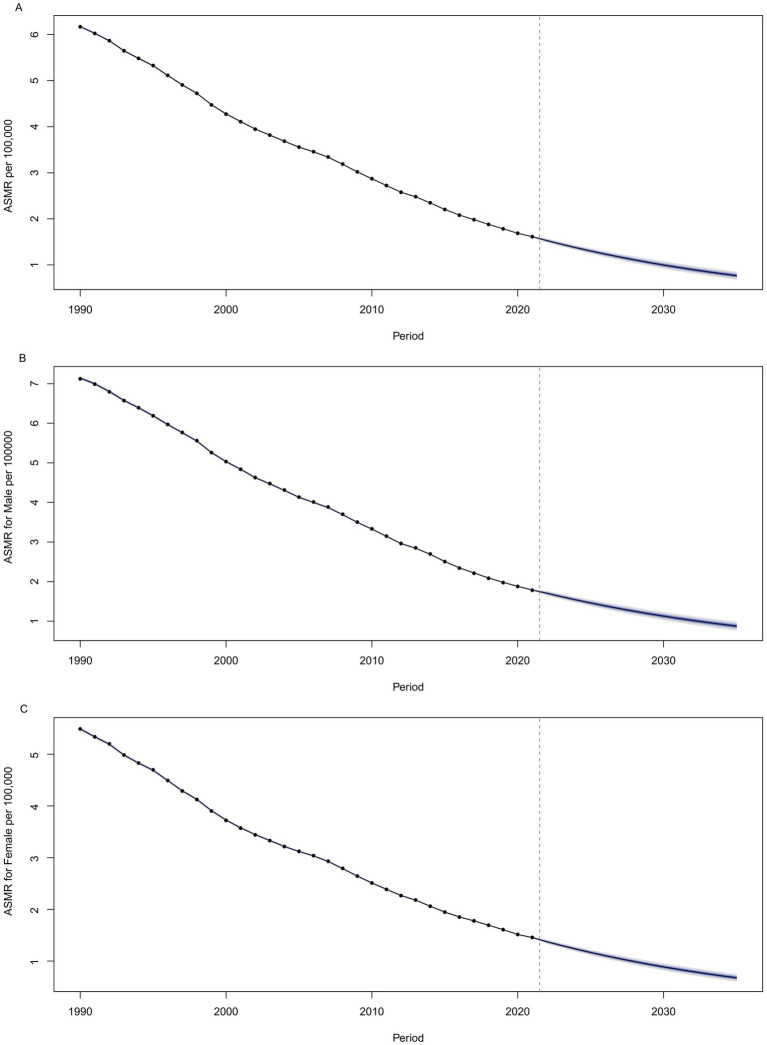
Projected values of global ASMR of NADs by 2035, **(A)** for both sexes, **(B)** for males, and **(C)** for females. NADs, Norovirus-Associated Diseases; ASMR, age-standardized mortality rate.

Globally, the ASMR for NADs is projected to continue its decline through 2035. By that year, the ASMR is anticipated to reach 0.88 per 100,000 for males and 0.68 per 100,000 for females, resulting in a combined rate of 0.77 per 100,000 for both genders. These estimates are illustrated in Figure 7 and detailed in Supplementary Table S5.

## 4 Discussion

### 4.1 Trend analysis and global health impact

Norovirus remains a significant cause of gastroenteritis outbreaks worldwide (25), primarily transmitted via the fecal-oral route (26). From 1990 to 2021, ASMR and ASDR for NADs have shown a significant decline across most regions, supported by a statistically significant EAPC (27), indicating a reduction in the global health burden of NADs (12). The mortality rate has dropped to a quarter of the 1990 levels, possibly due to enhanced public health measures and improved hygiene (28).

The GII.4 genotype’s predominance in the U.S., Europe, and Oceania between 1998 and 2007 led to increased hospitalization and mortality rates (11, 27). In contrast, in low SDI regions like Bangladesh, the GII.7 genotype was most predominant from 2014 to 2021 (29, 30). The global NADs mortality rate saw the most rapid decline between 2011 and 2014, coinciding with the establishment of NoroSTAT by the CDC and non-pharmaceutical interventions during the COVID-19 pandemic (31).

Diagnostic techniques for norovirus have advanced from traditional cell culture to high-speed, high-sensitivity molecular diagnostics, aiding in rapid diagnosis and effective outbreak management (32). However, for most of the study period, norovirus testing was not conducted; GI and GII genogroups were identified through RT-PCR testing of patients with watery diarrhea (15).

The GBD study derived its figures by multiplying the total cases of diarrhea by the estimated proportion of diarrhea cases attributable to noroviruses (33). This reduction in norovirus infections is closely linked to the decrease in global diarrhea cases, particularly evident in the 47.0% reduction in DALY rates for diarrhea from 2010 to 2021 (34). These estimates are influenced by GBD assessment coefficients.

The GBD’s counterfactual approach, which calculates PAF for each pathogen, assumes that the proportion of severe diarrhea cases positive for a specific pathogen indicates the mortality reduction if that pathogen were eliminated. This approach may not always be valid due to multiple pathogen interactions and co-infections. Therefore, the decline in diarrheal diseases does not necessarily reflect a decrease in all pathogens’ incidence. Caution is needed when interpreting GBD’s estimates, considering the methods’ limitations. Future research should refine these estimates with detailed epidemiological data and explore each pathogen’s dynamics in the context of global health improvements.

### 4.2 Geographical and socioeconomic disparities

The burden of norovirus extends across nations with varying income levels, with detection in all age groups in low-and middle-income countries, indicating the global distribution of the disease (35). It is crucial to consider the impact of norovirus on global health inequalities, as there are significant disparities in the burden of NADs among regions and countries with different SDI levels. Higher SDI correlates with lower disease burden; however, countries with lower SDI experience a more rapid reduction in disease burden compared to those with higher SDI. Apart from certain regions such as Western Europe, high-income North America, and Sweden, where an upward trend in mortality and disease burden is observed, most areas and countries show a downward trend in ASMR and ASDR, with the most significant declines in Central Asia and East Asia. These differences may be attributed to factors such as access to healthcare, urbanization, and education levels (17). Despite the higher disease burden in low SDI countries, the faster rate of decline may be associated with global health assistance and intervention measures (19).

Compared to developed countries, the incidence of NADs may be higher in developing countries, with South Asia and Africa bearing the heaviest disease burden (36). In settings with lower socioeconomic status, the high mortality rate from diarrheal diseases is caused by multiple factors, including malnutrition, poor water quality, inadequate access to healthcare, reduced diagnostic capacity, and poor disease management due to insufficient oral rehydration and zinc supplementation (11). New norovirus strains may emerge from children in developing countries and potentially lead to outbreaks spreading across continents. However, genetic characteristics may vary by region, impacting strain diversity and transmission dynamics (37). Norovirus continues to pose a significant threat in developing countries, leading to a substantial number of diarrheal deaths among children under 5 (38). In contrast, in developed countries, norovirus outbreaks are more likely to occur in healthcare settings among the older adult (1).

### 4.3 Disease burden in vulnerable populations

Norovirus is globally distributed and not confined by economic status. It is detectable in all age groups across low-and middle-income countries, demonstrating its widespread transmission globally (39). Our study identifies children and the older adult as populations requiring focused attention. Particularly in developing countries, the high transmission rate among children leads to repeated asymptomatic infections (40). High-risk groups that warrant attention include immunocompromised patients (41), hospitalized children (40), and older adult individuals in nursing homes (42). These groups are more susceptible to severe complications associated with norovirus, necessitating targeted preventive measures and vigilant surveillance to prevent nosocomial transmission and protect vulnerable pediatric populations. Children under the age of 5 deserve particular attention (43). The higher disease burden in these groups may be associated with biological, social, and environmental factors, such as the underdeveloped immune systems in children and the potential for immunosenescence and multimorbidity in the older adult (44). Preventive and control measures for these vulnerable populations, including vaccination and infection control strategies, are crucial for reducing their disease burden (45).

### 4.4 Future trends and policy recommendations

Based on the BAPC model’s predictions, NADs’ future trends indicate a continued decline in ASMR and ASDR by 2035. Despite the annual decrease in ASMR and ASDR for NADs, the viruses remain highly contagious, as even minimal viral particles can cause disease, and infected individuals shed a large quantity of the virus (39). The GII genotype is detected more frequently, accounting for 82.6% of positive cases, with GII.2 and GII.4 strains remaining prevalent, and the prevalence of GII.2 continues to increase (2). Norovirus remains a key cause of gastroenteritis, leading to school closures, loss of workdays, and significant impacts on public health (29).

We optimistically believe that the impact of norovirus can be improved through developing and implementing vaccinations for high-risk populations (45), enforcing strict infection control measures in healthcare facilities and nursing homes (46), enhancing surveillance systems for early detection and rapid response to norovirus outbreaks to prevent further spread (47), and continuing research on the epidemiology, genotypes, and transmission dynamics of norovirus (1). The real-time availability of Google Trends data can supplement existing norovirus surveillance (48), and investing in healthcare infrastructure in low-income countries will improve access to medical resources, clean water, and sanitation facilities (28, 49).

#### 4.4.1 Novelty

This study conducts a comprehensive, regional, and multi-faceted analysis to thoroughly examine the changes in the impact of NADs from 1990 to 2021 and predicts future burden changes up to 2035. Strengthening preventive actions for high-risk groups is crucial, especially for children under five and the older adult over 95. Understanding the epidemiological patterns and transmission of norovirus is critical for developing effective public health strategies aimed at minimizing the impact of NADs.

#### 4.4.2 Limitations

The accuracy of secondary research data is not very high. We must acknowledge that norovirus testing was not widespread for most of the study period. Upon reviewing the original data on norovirus used in the Global Burden of Disease Study (GBDS), we found that the data collection methods varied, making it difficult to calculate data accuracy. Additionally, the database only includes mortality and DALYs rates for NADs. As we understand, DALYs comprise YLL (Years of Life Lost) and YLD (Years Lived with Disability), but norovirus is unlikely to cause disability. These results suggest limitations in extrapolating the broader global mortality rate of norovirus.

#### 4.4.3 Ethical considerations

For the use of de-identified data in the GBD study, a waiver of informed consent has been approved by the Institutional Review Board of the University of Washington.

## Data Availability

The original contributions presented in the study are included in the article/Supplementary material, further inquiries can be directed to the corresponding author.

## Footnotes

^1^ http://ghdx.healthdata.org

^2^ https://ghdx.healthdata.org/search/site/norovirus

^3^ https://ghdx.healthdata.org/data-type/estimate

^4^ https://surveillance.cancer.gov/joinpoint/
