# Supplementary figures and images for: Global burden and trends of norovirus-associated diseases from 1990 to 2021 an observational trend study
Source: Front Public Health. 2025 Jan 7;12:1483149. doi: 10.3389/fpubh.2024.1483149 (PMC11747034; doi:10.3389/fpubh.2024.1483149)

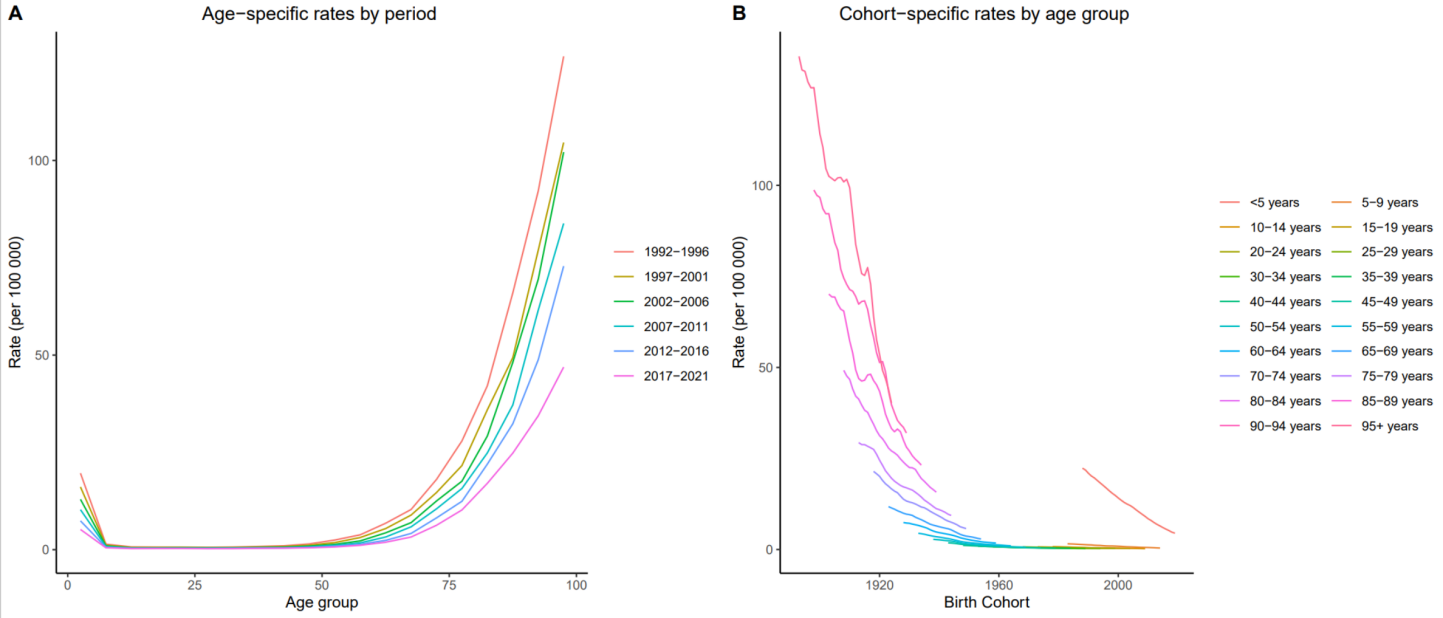

Supplement: Supplementary file 1 [file Image_1.png]
